# Application of ^18^F-FDG PET/CT imaging in gallbladder inflammatory pseudotumor with elevated CA199: a case report and review of literature

**DOI:** 10.3389/fonc.2023.1136876

**Published:** 2023-06-05

**Authors:** Mingyan Shao, Rong Xu, Wanling Qi, Zhehuang Luo, Fengxiang Liao, Sisi Fan

**Affiliations:** ^1^ Department of Nuclear Medicine, Jiangxi Provincial People’s Hospital, The First Affiliated Hospital of Nanchang Medical College, Nanchang, Jiangxi, China; ^2^ Department of Pathology, Jiangxi Provincial People’s Hospital, The First Affiliated Hospital of Nanchang Medical College, Nanchang, Jiangxi, China

**Keywords:** ^18^F-FDG PET/CT, gallbladder inflammatory pseudotumor, CA199, MRI, CT

## Abstract

**Background:**

Gallbladder inflammatory pseudotumor (GIPT) is a nonspecific chronic proliferative inflammation of the gallbladder. At present, the pathogenesis is not clear, which may be related to bacterial and viral infections, congenital diseases, gallstones, chronic cholangitis and so on. GIPT is rare and the imaging examination has no obvious specificity. There are few reports on the ^18^F-FDG PET/CT imaging characteristics of GIPT. In this paper, ^18^F-FDG PET/CT findings of GIPT with elevated CA199 are reported and the literature is reviewed.

**Case description:**

A 69-year-old female patient presented with recurrent intermittent right upper abdominal pain for more than 1 year, followed by nausea and vomiting for 3 hours, without fever, dizziness, chest tightness and other symptoms. Complete CT, MRI, PET/CT and related laboratory tests, CEA (-), AFP (-), Ca199 224.50U/mL ↑,^18^F-FDG PET/CT images showed uneven thickening at the bottom of the gallbladder, slightly increased gallbladder volume, eccentric and localized thickening of the gallbladder body wall, nodular soft tissue density shadow, clear boundary, smooth gallbladder wall, presence and smooth hepatobiliary interface, increased FDG radioactivity uptake, SUVmax was 10.2.The tumor was resected after operation and was diagnosed as gallbladder inflammatory pseudotumor by postoperative pathology.

**Conclusion:**

^18^F-FDGPET/CT imaging has a certain significance for gallbladder inflammatory pseudotumor. In patients with chronic cholecystitis, when the CA199 increases, the gallbladder wall appears localized thickening, the hepatobiliary interface exists and is smooth, and the ^18^F-FDG metabolism is mildly to moderately increase. Gallbladder cancer cannot be diagnosed alone, and the possibility of gallbladder inflammatory pseudotumor should also be considered. However, it should be noted that the cases with unclear diagnosis should still be actively treated with surgery, so as not to delay the treatment opportunity.

## Introduction

Inflammatory pseudotumor (IPT) is a kind of unexplained disease characterized by fibrous connective tissue hyperplasia with massive inflammatory cell infiltration. The disease can occur in any part of the body, most often in the lungs, but also in the central nervous system, liver, gallbladder, spleen, lymph nodes and other places (1, 2). It is not considered to be a true tumor with borderline biological behavior (3). Gallbladder inflammatory pseudotumor (GIPT) is rare, and its pathogenesis is still unclear. It may be related to viral or bacterial infection, congenital diseases, gallstones and chronic cholangitis, etc (4). GIPT is difficult to diagnose, and traditional imaging examination has no obvious specificity. Its imaging manifestations are mostly reported as individual cases, and there are fewer reports about its PET/CT imaging characteristics. This article reviews the PET/CT findings and diagnosis of gallbladder inflammatory pseudotumor with elevated CA199.

## Case description

A 69-year-old female patient presented with recurrent intermittent right upper abdominal pain for more than 1 year, which recurred for 3 hours. One year ago, she had intermittent right upper abdominal pain and discomfort without obvious inducement, no radiating pain in other parts. She was fearless of cold, fever, nausea and vomiting, headache and dizziness, etc. During this period, she was given anti-infection, liver protection and other support symptomatic treatment, and her symptoms were slightly relieved. The patient developed pain and discomfort in the right upper abdomen again without obvious inducement 3 hours ago, accompanied by nausea and vomiting, without fever, dizziness, chest tightness and other symptoms.

**Figure 1:**
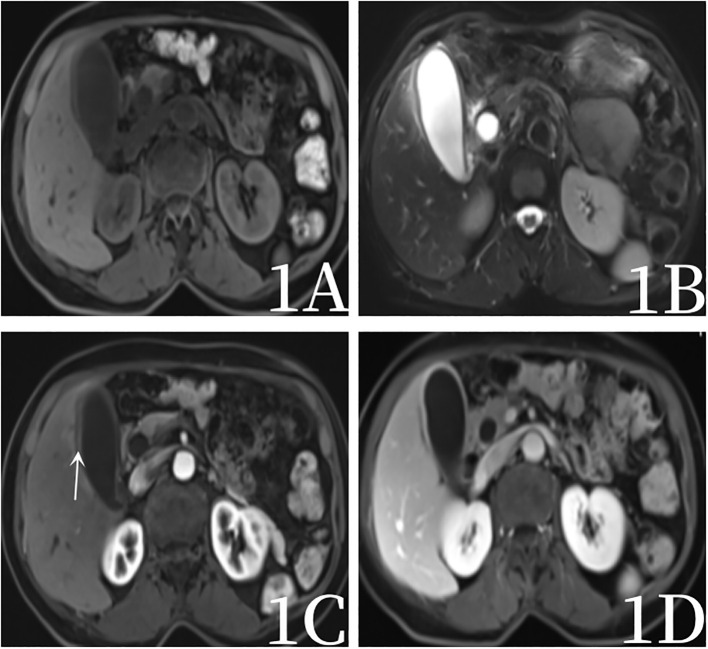
Female, 69 years old, a year ago, magnetic resonance examination, 1**A** (T1-weighted images), 1**B** T2-weighted images), 1**C** (arterial stage 20s), 1**D** (portal stage 55s) showed a slight thickening of the gallbladder wall (arrow), mild enhancement, gallbladder sediment stones, no signs of gallbladder tumor.

**Figure 2:**
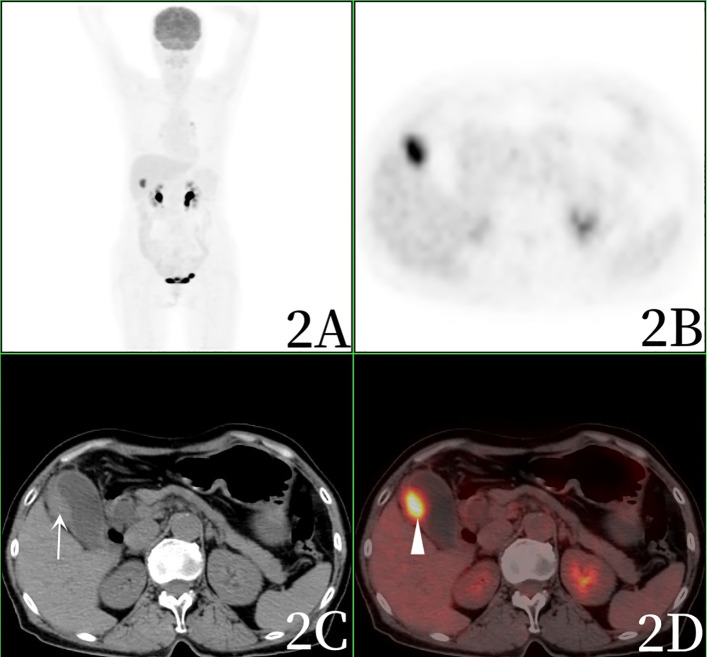
Female, 69 years old.1h after injection of 18F-FDG into PET/CT, 2**A** (whole body MIP), 2**B** (axial PET), 2**C** (axial CT), 2**D** (axial fusion) showed slightly increased gallbladder volume, uneven thickening of gallbladder bottom, eccentric and limited thickening of gallbladder body wall (arrow), and nodular soft tissue density shadow with a size of about 13×19mm. The hepatobiliary interface was clear, radioactivity uptake increased, and SUVmax was 10.2 (arrow).

**Figure 3:**
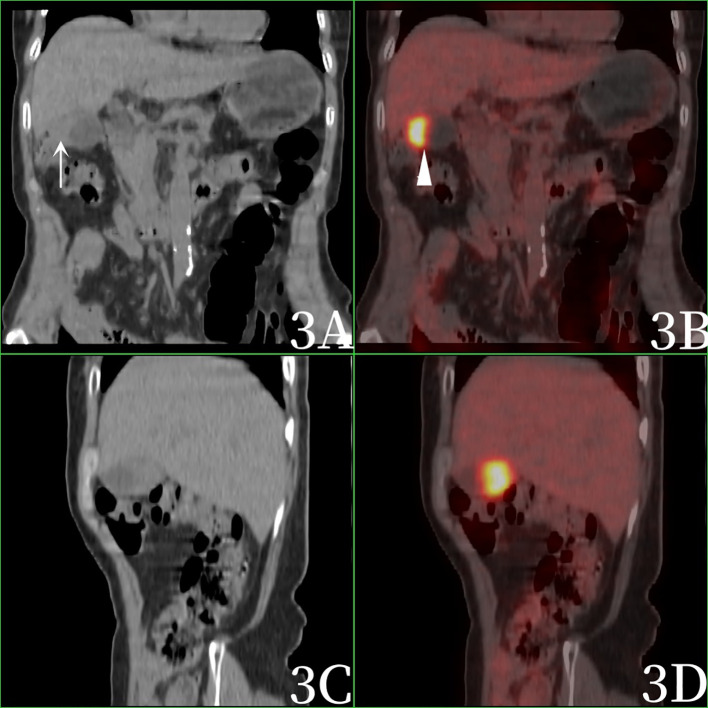
Female, 69 years old. 3**A** (coronal CT), 3**B** (coronal fusion), 3**C** (sagittal CT), and 3**D** (sagittal fusion). Imaging was performed 1h after 18F-FDG injection. PET/CT showed eccentric and localized thickening of the gallbladder body wall (arrow), nodular soft tissue density shadow, increased radioactive uptake, and SUVmax was 10.2 (arrow).

After admission, the blood routine test report showed that the percentage of lymphocytes was 16.9%↓, the percentage of neutrophils was 77.7%↑, and the other blood routine indicators were normal. Blood biochemical test report: Total protein 64.9g/L↓, albumin 37.8g/L↓, Alanine aminotransferase 174IU/L↑, Aspartate aminotransferase 420IU/L↑, glutamyltransferase 170IU/L↑, alkaline phosphatase 166IU/L↑, glucose 7.3mmol/L↑, lactate dehydrogenase 435IU/L↑, Creatine kinase isoenzyme 29IU/L↑, angiotensin converting enzyme 62.3U/L↑.Blood tumor marker report: CEA (-), AFP (-), CA199 224.50U/mL ↑.One year ago, the patient underwent magnetic resonance imaging (MRI)examination of the upper abdomen due to abdominal pain, and MRI showed chronic cholecystitis without gallbladder tumor signs (**Figure 1**). After admission, computed tomography(CT) scan showed increased gallbladder volume, uneven density at the bottom of the gallbladder, fusiform thickening of the gallbladder wall, uneven thickness, and rough edges, suggesting gallbladder neoplastic lesions and high possibility of gallbladder cancer. The patient was examined directly by PET/CT without MRI, PET/CT showed uneven thickening at the bottom of the gallbladder, slightly increased gallbladder volume, and eccentric and localized thickening of the gallbladder body wall, PET/CT showed nodular soft tissue density shadow, about 13×19mm in size, increased radioactive uptake, SUVmax was 10.2, which was considered to be a high possibility of gallbladder cancer. PET/CT also revealed stenosis of the lower common bile duct, which was considered to be inflammatory (**Figures 2**, **3**).

**Figure 4:**
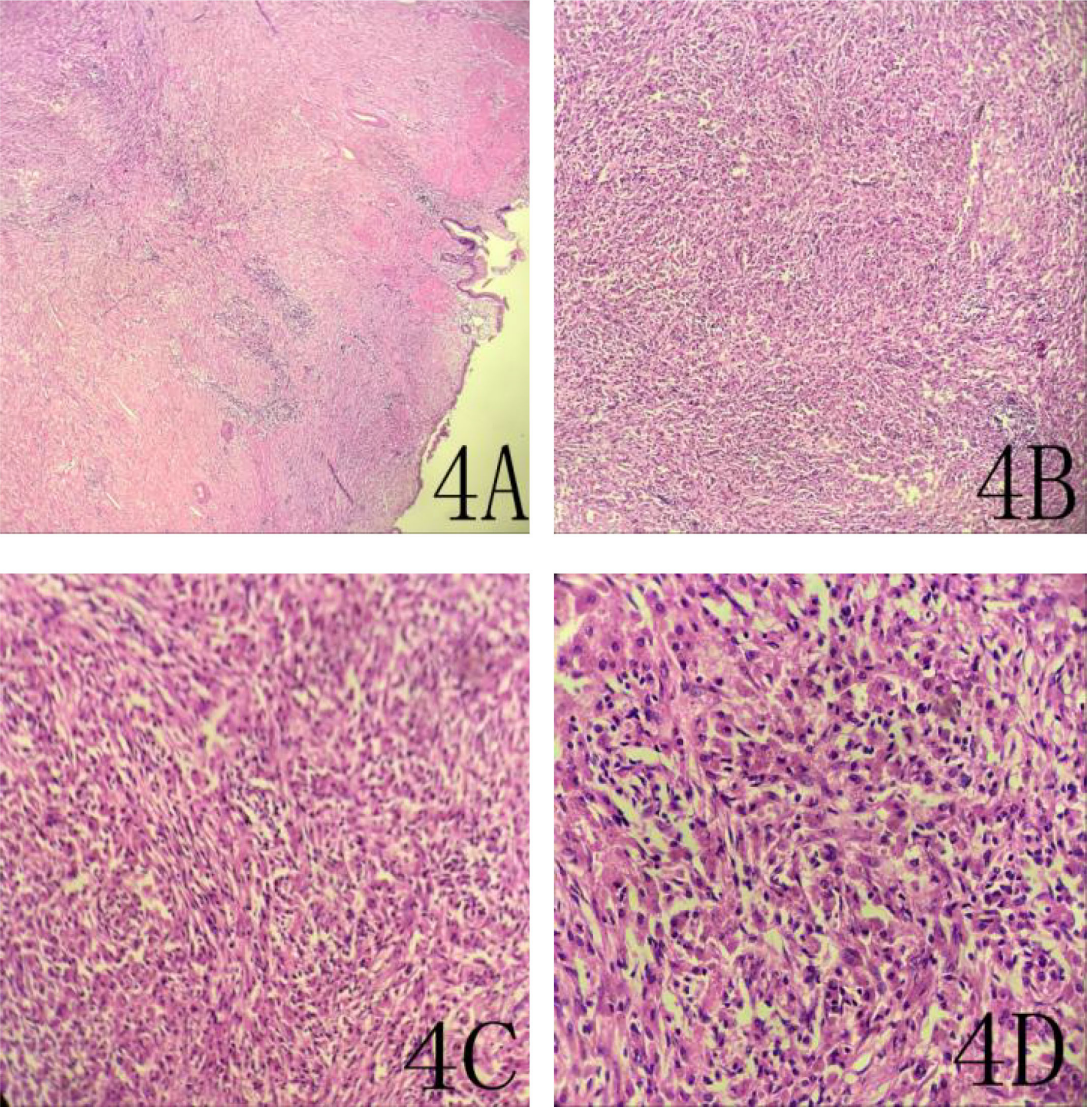
A 69-year-old woman with an inflammatory pseudotumor of the gallbladder. 4**A** (HE×40), 4**B** (HE×100), 4**C** (HE×200), 4**D** (HE ×400).This image shows localized fibrous connective tissue hyperplasia with extensive chronic inflammatory cell infiltration.

The patient was considered to have malignant tumor before surgery, and planned to undergo laparoscopic cholecystectomy (radical resection of gallbladder cancer) and cholangio-jejunostomy. Intraoperative inspection: One cholecystectomy specimen, 7×4.5×3.8cm in size, contained dark green bile, the focal folds of the mucosal surface disappeared, accompanied by hard gallbladder wall, the range was about 2.5×2cm, and the section of the gallbladder wall showed pale yellow deposition, medium and solid, and the thickness of the gallbladder wall was about 0.2-1.3cm.Frozen pathology showed spindle cell tumor (gallbladder), tending to borderline tumor, with no tumor involvement at the surgical margin. Therefore, we decided to perform hilar lymph node dissection, dissociate the common hepatic artery and proper hepatic artery, and dissect the 8th group of lymph nodes. The common bile duct and portal vein were separated, and the 12th group of lymph nodes were dissected until the common bile duct, main portal vein, common hepatic artery, and proper hepatic artery were nude. Finally, pathology suggested inflammatory pseudotumor of the gallbladder (**Figure 4**), Reactive hyperplasia of lymph nodes in group 12A, group 12C, and group 12P.The patient recovered well after surgery and had no significant signs of recurrence after one-year of follow-up.

## Discussion

Gallbladder inflammatory pseudotumor is a kind of nonspecific chronic proliferative inflammation of the gallbladder, which can occur at any age, most of which are middle-aged people (5). GIPT reports are rare and easily misdiagnosed as gallbladder cancer. This case was misdiagnosed as gallbladder cancer before surgery, and was diagnosed as GIPT by postoperative pathology.

At present, the pathogenesis of GIPT is still unclear, which may be related to viral or bacterial infection, congenital diseases, gallstones and chronic cholangitis. Imaging examination of GIPT is not specific (6). GIPT was found for the first time in more cases by ultrasound, which may be due to the convenience and popularity of ultrasound. On the ultrasound imaging, GIPT may present as hyperechoic, hypoechoic or mixed echogenic occupation of the gallbladder with blurred boundaries. GIPT showed fast-in and fast-out enhancement under contrast-enhanced ultrasound in most cases. Although most cases showed enhancement after contrast injection, the degree of enhancement was not very high, with mild high or moderate enhancement in arterial phase and low enhancement in portal phase and delayed phase, which may be due to the fact that the lesions were mostly inflammatory vessels (2, 6). The CT plain scan disclosed lumpy and lumpy soft tissue shadows with unclear boundaries, and the surrounding gallbladder wall was thickened, with mild enhancement. The reason may be that there were more fibrous tissues and vascular hyperplasia in such lesions, which could not be quickly removed when the contrast agent entered the extravascular space (7, 8). On MRI,T1-weighted images can show low signal, isosignal or high signal,T2-weighted images show high signal, which may be related to liquefaction and necrosis in the lesion. Most of the enhanced images show no enhancement in the arterial phase, and the portal phase shows circular enhancement, which continues to the delayed phase. Delayed enhancement of GIPT in portal phase and delayed phase is of great significance for the diagnosis of inflammatory pseudotumor (9).

As a functional metabolic imaging, PET/CT can diagnose the benign and malignant tumors through the metabolic information of ^18^F-FDG. PET/CT has the advantages of both CT and PET, which can not only show the anatomical images of the tomography, but also show the metabolic advantages of PET. The PET/CT images of GIPT showed masses and bars in the body and bottom of the gallbladder, increased FDG uptake was generally mild and moderate, and the uptake value was lower than that of malignant tumors. This overlaps with the metabolic manifestations of gallbladder malignancy, which easily leads to misdiagnosis. The reason for this is that GIPT is not a real tumor, it is mostly localized fibrous connective tissue hyperplasia with a large number of chronic inflammatory cell infiltration (10–12). There are mainly fibrous tissue and inflammatory cells, including plasma cells and lymphocytes in the histological manifestations. Since the expression of glucose transporters in malignant tumors, infectious and non-infectious inflammatory lesions is up-regulated compared with normal cells, and the hexokinase activity is higher than that in normal cells, all activated abnormal cells such as granulocytes, monocytes and lymphocytes will also take up ^18^F-FDG in large quantities, making ^18^F-FDG accumulate in the lesions where leukocytes accumulate (13). Therefore, GIPT can show abnormal hypermetabolic zone in ^18^F-FDGPET/CT imaging, which is easy to be misdiagnosed as gallbladder cancer. Although ^18^F-FDGPET/CT imaging is not specific for gallbladder inflammatory pseudotumor, it can show the extent of lesion and metabolic activity in other parts of the body, which can guide clinical treatment.

The patient with elevated CA199 and increased FDG metabolism in the lesion was more inclined to diagnose gallbladder cancer before surgery. The tumor marker CA199 of GIPT can be elevated, and the tumor marker CA199 of gallbladder cancer can also be elevated. It is not feasible to distinguish the two by tumor markers. The tumor marker CA199 exists in normal human pancreas, gallbladder, liver, intestine, bile duct epithelium, salivary gland, breast, bronchial epithelial cells, endometrium and other tissues (14). When the tissue is diseased, especially when the malignant tumor is diseased, the secretion is hypersecreted and released into the blood through the tumor vessels, resulting in hyperCA199emia, which is mainly seen in pancreatic cancer, hepatobiliary cancer, gastric cancer, colorectal cancer, ovarian cancer, liver cancer, esophageal cancer and lymphoma (15). Some benign diseases can also appear transient, low concentration increase, mainly in acute and chronic pancreatitis, cholelithiasis, liver cirrhosis, renal insufficiency, diabetes, etc (16). The patient’s clinical symptoms, imaging examination, and increased tumor markers increased the probability of misdiagnosis. Because to these factors, the patient finally chose surgical treatment and was finally diagnosed as inflammatory pseudotumor of the gallbladder. The preoperative diagnosis of GIPT is difficult, and it is most important to distinguish GIPT from gallbladder carcinoma, inflammatory myofibroblastic tumor and xanthogranulomatous inflammation. Gallbladder carcinoma is the most common clinical malignancy, which originates from epithelial tissue and is characterized by invasion of mucous membrane. It is often manifested as the inner wall of the gallbladder is not only integrated, the mucosal line is often incomplete or disappeared, the thickened gallbladder wall is easy to form a mass, often invading the surrounding liver, resulting in blurred or even disappeared hepaticiliary interface. Inflammatory myofibroblastic tumor of the gallbladder is very rare clinically, and the onset is more insidious. The clinical manifestations are mostly caused by the mass itself and the compression of the surrounding organs, and also have fever, anemia, fatigue, weight loss, abdominal pain and so on. The clinical presentation is similar to malignancy but lacks specificity and symptoms and signs disappeared after resection of the lesion. The diagnosis of this disease depends on pathological examination. Xanthogranulomatous inflammation is common in clinic. It is a special type of gallbladder inflammatory disease characterized by xanthogranulomatous formation and severe proliferative fibrosis. It tends to occur in women aged 60-70 years and is characterized by limited or diffuse thickening of the gallbladder wall, with complete gallbladder mucosal line and sandwich biscuit sign. The sandwich biscuit sign refers to the enhancement of the inner and outer ring of the thickened gallbladder wall without the enhancement of the low density nodules in the middle. This is the characteristic manifestation of xanthogranulomatous inflammation. The presence of this sign can diagnose xanthogranulomatous inflammation.

At present, there is no optimal treatment plan for GIPT, and surgical treatment is mainly used in clinical practice. Conservative treatment can be chosen for cases with pathological diagnosis obtained by ultrasound or CT-guided needle biopsy before surgery. Zhou et al. (17) reported that the use of antibiotics, non-steroidal anti-inflammatory drugs, steroids and other drugs could make GIPT lesions subside, and some patients had natural remission or subside without any treatment. However, it should be noted that patients with unclear diagnosis or ineffective conservative treatment should be actively treated with surgery.

## Conclusions

The conventional imaging examination of GIPT has no specificity, ^18^F-FDG PET/CT imaging has a certain significance for gallbladder inflammatory pseudotumor. In patients with chronic cholecystitis, when CA199 increases, the gallbladder wall appears localized thickening, the gallbladder wall is still smooth, the hepatobiliary interface exists, and the PET/CT metabolism is mildly to moderately increase. Gallbladder cancer cannot be diagnosed alone, and the possibility of gallbladder inflammatory pseudotumor should also be considered. However, it should be noted that the cases with unclear diagnosis should still be actively treated with surgery, so as not to delay the treatment opportunity.

## Data availability statement

The original contributions presented in the study are included in the article/supplementary material. Further inquiries can be directed to the corresponding author.

## Ethics Statement

Written informed consent was obtained from the participant/patient(s) for the publication of this case report.

## Author contributions 

MS, the first author, directed the operation and wrote the paper. RX, WQ, ZL, have participated in the design of the report and copy edited the manuscript. SF, has made the pathological diagnosis and part of the literature review. All authors read and approved the final version of the manuscript. All authors of this manuscript are in agreement with its content and are not being published or under consideration in another scientific journal. All authors contributed to the article and approved the submitted version.
